# Genomics of Fungal Disease Resistance in Tomato

**DOI:** 10.2174/138920210790217927

**Published:** 2010-03

**Authors:** Dilip R. Panthee, Feng Chen

**Affiliations:** 1 Department of Horticultural Science, North Carolina State University, Mountain Horticultural Crops Research and Extension Center, 455 Research Dr., Mills River, NC 28759, USA; 2 Department of Plant Sciences, University of Tennessee, Knoxville, TN 37996, USA

**Keywords:** Comparative genomics, functional genomics, genomics, QTL analysis, *Solanum lycopersicum*, tomato.

## Abstract

Tomato (*Solanum lycopersicum*) is an important vegetable crop worldwide. Often times, its production is hindered by fungal diseases. Important fungal diseases limiting tomato production are late blight, caused by *Phytophthora infestans*, early blight, caused by *Alternaria solanii*, and septoria leaf spot, caused by *Septoria lycopersici*, fusarium wilt caused by *Fusarium oxysporium fsp. oxysporium, *and verticilium wilt caused by *Verticilium dahlea*. The *Phytophthora infestans *is the same fungus that caused the devastating loss of potato in Europe in 1845. A similar magnitude of crop loss in tomato has not occurred but *Phytophthora infestans *has caused the complete loss of tomato crops around the world on a small scale. Several attempts have been made through conventional breeding and the molecular biological approaches to understand the biology of host-pathogen interaction so that the disease can be managed and crop loss prevented. In this review, we present a comprehensive analysis of information produced by molecular genetic and genomic experiments on host-pathogen interactions of late blight, early blight, septoria leaf spot, verticilim wilt and fusarium wilt in tomato. Furthermore, approaches adopted to manage these diseases in tomato including genetic transformation are presented. Attempts made to link molecular markers with putative genes and their use in crop improvement are discussed.

## INTRODUCTION

Tomato (*Solanum lycopersicum*, formerly, L*ycopersicon esculentum* Mill.) is the second most important vegetable crop after potato in the world. In addition to being consumed as a fresh vegetable, it is also used as a salad, in ketchup, as a puree, a pickle and in many other forms, depending up on the growing area. It is estimated that 4.6 million ha of tomatoes are grown annually worldwide producing more than 126 million mt. In the U.S., it is grown in an area of 175,000 ha producing about 11.5 million mt. annually (http://faostat.fao.org/). In addition to being an important vegetable crop worldwide, tomato is also used as a model plant species for genetic studies related to fruit quality, stress tolerance (biotic and abiotic) and other physiological traits. It is widely adapted to a variety of climates spanning the tropics to temperate regions. In order to meet the demand for tomatoes, it is also grown in greenhouses. Because of its economic contribution to the agriculture industry, there is abundant interest in using genomic tools to improve tomato and develop new varieties.

Despite decades of conventional breeding and selection, there are still a large number of fungal diseases that make tomato production challenging in various parts of the world. Current advances in plant genomics, including structural and functional genomics and in biotechnology provide important tools for tomato improvement [1]. These tools are being used for genetic analysis and crop improvement in a number of crop plants including rice [2, 3], wheat [4], brassica [5], maize [6], soybean [7], cotton [8] and vegetable crops [1, 9]. In this review, we will discuss the use of relatively new tools for understanding and improving disease resistance in tomato. 

Major fungal diseases of tomato posing a threat in tomato production are late blight, early blight, septoria leaf spot, fusarium wilt and verticilium wilt. Other fungal diseases of tomato include powdery mildew caused by *Oidium lycopersicum* and leaf mold caused by *Cladosporium fulvum*. Fusarium and verticilium wilt are vascular diseases, while the first three are foliar diseases. Foliar diseases are more important and cause significant crop losses annually worldwide. Because prevailing weather conditions determine the severity of the disease, crop loss may range from mild loss of productivity to the complete loss of a crop. 

Late blight (LB) is the most important foliar fungal disease of tomato. It is caused by *Phytophthora infestans* (Mont.) de Bary and can result in severe crop damage. The disease is favored by cool temperatures and humid, rainy or foggy conditions. This pathogen can spread in a very short period of time since a single lesion can produce as many as 300,000 sporangia per day [10]. It is very difficult to detect *P. infestans *in the field during initial stages of infection, and because of its very short life cycle, by the time the disease becomes detectable it is already too late to protect the crop through fungicide application. The short life cycle of the disease makes the spread of infection rapid. The *P. infestans* genome has been sequenced and its functional genomics for virulence has been investigated [11]. This information may be helpful in developing new strategies to increase resistance in host plants.

Early blight (EB) is another important foliar fungal disease of tomato, and is caused by the necrophytic fungus *Alternaria solani* Jones and Grout. This is the most common disease of the cultivated tomato in areas with heavy dew, frequent rainfall, and high humidity [12]. The necrotrophic nature of the pathogen can lead to complete defoliation of tomato plants and subsequent yield reductions. Almost all member species of the Solanaceae can serve as alternate host for overwintering of the pathogen. 

Septoria leaf spot (SLS) (caused by *Septoria lycopersici* Speg) is yet another destructive foliar disease of tomato worldwide. Extended periods of wet and humid weather conditions are conducive for disease development. Circular lesions first appear on the lower leaves, thereafter appearing on stem, petioles and calyx. The disease appears after first fruiting and spreads upward. Powdery mildew (PM) is caused by *Oidium lycopersicum* and is also an important foliar disease of tomato [13]. In some reports, the causal agent of powdery mildew is also reported as *Erysiphe orontii*. The disease occurs during warm and dry seasons. Symptoms may progress from light green to bright yellow necrotic lesions and eventually a light powdery covering of the leaf surface of tomato, which may cause leaf death. 

Fusarium wilt (FW) (caused by *Fusarium oxysporum *Schlectend.) can cause severe losses in tomato. There are two distinct forms of the pathogen, *F. oxysporum *f. sp. *lycopersici *W. C. Snyder & H. N. Hans which causes vascular wilt, and *F. oxysporum *f. sp. *radicis-lycopersici* W. R. Jarvis & Shoemaker which causes crown and root rot. Both of these pathogens are soil borne and occur throughout most tomato growing areas [12]. Infected leaves start drooping, curve downwards and turn yellow. Disease symptoms are apparent during flowering and fruiting stages, and leaflets on one side of the plants typically show more severe symptoms than leaves on the other side because of the specific vascular tissue affected by the pathogen. Subsequently, plants start wilting during hot days and eventually die [14]. There are three races of this pathogen, race 1, 2 and 3, of which race 3 is the most devastating. Verticilium wilt (VW) caused by *Verticilium dahliae* is also a soil borne Ascomycete and like FW causes significant losses in tomato. VW has a wide host range and is distributed throughout the world. The fungus overwinters in plant debris and alternate hosts. Relatively cool temperatures, high humidity and high soil moisture are conducive to the spread of this disease [12]. Disease symptoms appear on the lower leaves as yellow blotches, wilting and eventually dropping off. There are two races of this fungus that are active in tomato, *Ve-1* and *Ve-2*. We will try to present the comprehensive genomic research on all these fungal diseases of tomato in this review.

## QTL AND MOLECULAR MARKERS FOR FUNGAL DISEASES

Since the Irish famine, there has been a lot of interest in late blight research, primarily in potato but also in tomato. Both species are severely affected by this crippling disease. As a result of much research, the dominant resistance gene *Ph-1* was identified in the wild relative of tomato *Solanum pimpinellifolium* (formerly *Lycopersicon pimpinellifolium*) and was mapped to the distal end of the chromosome 7 [cited in 10]. However, this gene was not effective for long time due to the emergence of new race of *P. infestans*. Subsequently, a partial dominant gene *Ph-2* was found in the same wild relative *S. pimpinellifolium*, that mapped to chromosome 10 [15]. Molecular markers TP105 and TG233 were found to be closely associated with *Ph-2*. Again, the resistance conferred by this gene was found to be ineffective in the long term and yet another resistance gene was isolated from *S. pimpinellifolium* named *Ph-3*, which mapped to chromosome 9 [16]. Although molecular marker TG591A is closely associated with *Ph-3, *there are not any reports utilizing this marker in a tomato breeding program, perhaps because it has to be fine-mapped before use in marker-assisted selection. 

In addition to this, there are a few reports of quantitative resistance to late blight and the QTLs associated with it. In one study, reciprocal backcross populations derived from *Solanum lycopersicum *× *Solanum* *habrochaites* (formerly, *Lycopersicon hirsutum) *(population BC-E was the backcross to *S. lycopersicum*, and BC-H was the backcross to *S. habrochaites*) were assessed using three types of replicated disease assays (detached-leaflet, whole-plant, and field) [17]. Linkage maps were constructed for each BC population using RFLPs. Resistance QTLs were identified on all 12 tomato chromosomes using composite interval mapping. Six QTLs in BC-E (*lb1a*, *lb2a*, *lb3*, *lb4*, *lb5b*, and *lb11b*) and two QTLs in BC-H (*lb5ab *and *lb6ab*) were most consistently detected in replicated experiments and across assay methods [17]. *S. habrochaites *alleles conferred resistance at all QTLs except *lb2a*. Resistance QTLs coincided with QTLs for inoculums droplet dispersal on leaves, a trait in *S. habrochaites *that may contribute to resistance. Not surprisingly the dispersal QTL was mainly associated with leaf resistance [17]. Some *P. infestans *resistance QTLs detected in tomato coincided with chromosomal locations of previously mapped R genes and QTLs in potato for resistance to *P. infestans*, suggesting functional conservation of resistance within the *Solanaceae*.

These QTLs were then fine mapped and verified using near-isogenic lines (NILs) for* lb4*,* lb5b*, and* lb11b* by marker-assisted backcrossing to* S. lycopersicum*. Sub-NILs containing overlapping* S. habrochaites *segments across each QTL region were selected and used to fine-map and validate the QTL effects [18]. The NILs and sub-NILs were evaluated for disease resistance at three field locations. Resistance QTLs were detected in all three sets of NILs, confirming the BC_1_ mapping results.* Lb4* mapped near TG609, and between TG182 and CT194, on chromosome 4 at an interval of 6.9-cM. *Lb5b* mapped to an 8.8-cM interval between TG69a and TG413 on chromosome 5, with the most likely position near TG23, and* lb11b* mapped to a 15.1-cM interval on chromosome 11 between TG194 and TG400, with the peak centered between CT182 and TG147 [18]. Fine mapping of these QTLs made potential MAS for LB resistance.

Subsequently, two populations of *Solanum pennellii* (formerly, *Lycopersicon pennellii*) were screened for resistance to LB. Resistance was identified through assessment of disease progress in an F_2_ mapping population derived from *S. lycopersicum *× *S. pennellii *crosses. Levels of resistance varied widely among individuals within each population. However, the response of individuals to different strains of *P. infestans* was consistent. A resistance QTL that accounted for 25% of the phenotypic variation in the population was detected near marker T1556 on chromosome 6 [19]. The occurrence of this QTL was confirmed from analysis of the introgression lines. Another resistance QTL was also found on the same chromosome 6 [17].

Early blight (EB) resistance is a quantitative trait, which makes selection more difficult compared to qualitative traits. Major sources of resistance for EB have been identified in *S. habrochaites, S. pimpinellifolium* and *S. peruvianum. *An accession PI138630 from cultivated tomato, however, has been the major source of resistant genes used in developing early blight resistant lines in a number of breeding programs [see 10]. In order to understand the genetic control of early blight resistance and to facilitate its introgression in tomato, molecular markers and QTL analysis has been carried out . One of the most extensive studies to date was performed by Foolad *et al*. [20]. They used a backcross population derived from a susceptible tomato breeding line NC84173 and a resistant *S. habrochaites* accession PI126445 to map resistance QTL for EB. They genotyped 145 plants with 141 RFLP markers and 23 resistance gene analogs (RGA), and a genetic linkage map was constructed. The BC_1_ plants were selfed to produce a BC_1_S_1_ mapping population. They found ten resistance QTL for EB in both BC_1_ and BC_1_S_1_ populations, which were highly consistent across generations, and years. Individual QTL explained 8.4% to 25.9% of total phenotypic variation whereas the combined effect was more than 57% [20]. Unlike other QTL analysis studies, all resistance QTL alleles were contributed by the resistant parent *S. habrochaites*. Close agreement between two years and two generations indicated the stability of the identified QTLs and their potential usefulness for improving tomato EB resistance using MAS. However, specific markers have not yet been employed in any selections. 

Later, a selective genotyping approach was used to validate the resistant QTLs detected using PI126445 of *S. habrochaites *[21]. A total of 820 BC_1_ plants from a cross between an EB susceptible tomato breeding line NC84173 and PI126445 were grown in a greenhouse. Nine weeks old plants were inoculated with two isolates of *A. solani* and evaluated for EB symptoms. Both the most resistant and the most susceptible plants were selected and subsequently transplanted into a field where natural infestation of EB was severe. The 76 selected plants, representing the two extremes of the response distribution, were genotyped with 145 RFLP markers and 34 RGA. A trait-based marker analysis detected seven QTLs for EB resistance, four of which had been detected in a previous study [21]. 

In order to identify and estimate the effect of genes conditioning resistance to EB, a QTL mapping study was performed in F_2_ and F_3_ populations derived from a cross between *S. lycopersicum* cv. Solentos (susceptible) and *Solanum peruvianum* LA2157 (resistant) [22]. They used 344 AFLP (with 12 primer combinations), 36 SSR and 14 SNP markers for genotyping 176 F_2_ individuals. A total of six QTL regions were mapped to chromosomes 1, 2, 5, 6, 7, and 9, including three resistance QTL to stem lesions in the field that explained 35% of the phenotypic variation [22]*.* All QTL displayed significant additive gene effects but the QTL on chromosome 9 was also found to have a dominance effect.

FW caused by *Fusarium oxysporum f. sp. lycopersici* (*Fol*) is a devastating disease of tomato [12]. Three races, race-1, race-2 and race-3, of *Fol* have been reported to cause this disease. Corresponding to these races, three loci *I-1, I-2* and *I-3*, have been identified which confer resistance in tomato. The locus *I-2* was introgressed into *S. lycopersicum* from the wild species *S. pimpinellifolium* accession PI126915. Available RFLPs were searched between near isogenic lines (NILs) that map to the region introgressed from the wild species. *I-2* mapped to chromosome 11, so DNA clones from this chromosome were used as hybridization probes to Southern blots containing DNA from the NILs. Of the 14 clones, 9 exhibited a polymorphism on chromosome 11 [23]. These clones were further hybridized to verification filters containing DNA from resistant and susceptible *S. lycopersicum *varieties digested with the enzymes that produced the polymorphism. One clone, TG105 was found to be associated with *I-2,* 19 susceptible lines showed a different RFLP with this probe from 16 resistant lines, including the original *S. pimpinellifolium* accession used as a source for the resistance gene. These results indicated that TG105 is closely linked to the resistance gene for *Fol* on chromosome 11 [23]. Subsequently, *I-2* was mapped between the RFLP markers TG105 and TG36, 0.4 cM from TG105 on the same chromosome [24]. They also generated new RFLP markers in the region by chromosome walking from TG105 toward *I-2.*

In addition to the above, resistance to different pathogenic races of *Fol* has been explored at the genomic level in tomato. Six independent FW resistance loci were identified by comparing the responses of a complete set of 53 lines carrying different introgressed regions of the *S. pennellii *genome in a *S. lycopersicum* background. The loci conferred varying degrees of resistance to different races of the pathogen [25]. Corresponding map positions from two tomato species were aligned and in some cases revealed parallel resistance to *Fol* with qualitative changes in race specificities. One of the loci identified corresponds to the previously characterized complex resistance locus *I-2*. A novel member of this locus,* I-2C-5*, belonging to the *NBS-LRR* family of resistance genes, was cloned and shown to confer partial resistance in transgenic plants [25]. Thus, at a particular complex locus, individual gene members can confer full or partial resistance to *Fol* race 2. The results of whole-genome mapping analysis underline the robust independent origin of resistance to a particular disease and demonstrate the conservation of resistance features at conserved DNA sequence (synteny) along the chromosome of different species, together with the rapid diversification of genes for innate resistance within loci.

The inheritance and linkage relationships of a gene for resistance to *Fol* race 1 were analyzed subsequently using a back-cross population derived from a resistant *S. pennellii *and a susceptible *S. lycopersicum*, and backcrossed to a *S. lycopersicum*. The genotype of each BC_1_ plant with respect to its FW response was determined by means of progeny tests. It was reported that resistance was controlled by a single dominant gene, *I-1*, which was not allelic to *I*, the traditional gene for resistance against this fungal pathogen that was previously identified in *S. pimpinellifolium*. Linkage analysis of 154 molecular markers that segregated in the BC_1_ population placed *I-1* between the RFLP markers *TG20* and *TG128* on chromosome 7 [26]. Flanking markers were used to verify the assignment of the *I-1* genotype in the segregating population. 

Eventually the* I-3* gene from wild tomato *S. pennellii *accessions LA716 and PI414773 that confers resistance to *Fol* race 3 was mapped to chromosome 7 [27]. In this study, the RFLP, conserved ortholog set (COS) markers, known genes and a RGA mapping to the* I-3* region were converted into PCR-based markers such as SCAR, CAPS and SSRs. Additional PCR-based markers were also generated using the randomly amplified DNA fingerprinting (RAF) technique and used for high-resolution mapping around the* I-3* locus. The* I-3* gene was localized to a 0.3-cM region containing a RAF marker eO6, and an RGA marker RGA332. Despite the presence of two *RGA332* homologues in* S. lycopersicum*, DNA blot and PCR analyses suggested that no other homologues were present in lines carrying* I-3* that could be alternative candidates for the gene [27]. Concurrently, a detailed review on mapping the *I-3* locus in tomato and the use of molecular markers associated with FW for tomato improvement is available elsewhere [28]. They have also constructed a high-resolution map of the *I-3* region and co-segregating markers were identified that closely flank *I-3*. They report that the *I-3* gene co-segregates with four markers, flanked by two markers upstream and one marker downstream, defining an estimated genetic interval of 0.57 cM. In addition, they also found that the *I-3* gene in *S. pennellii *accessions LA716 and PI1414773, but the one from PI1414773 was designated as *I-7*. 

In contrast to the fungal diseases discussed above, there is a lack of QTL and molecular markers knowledge for SLS, VW, PM and other fungal diseases of tomato. It should be noted that even if EB, LB and FW are not present in the field, SLS is still a significant problem in tomato, for which introgression of resistance genes using molecular approach is crucial. 

## TOMATO GENOMIC RESOURCES AND DATA MINING FOR DEFENSE GENES

Despite the progress made in QTL mapping and marker development for tomato fungal disease resistance described in the previous section, it has been difficult to elucidate the genetic basis of tomato resistance to microbial pathogens, mainly due to the low level of genetic polymorphism within *S. lycopersicum* [29]. Mining genomic sequences for novel markers, such as single nucleotide polymorphisms (SNPs) has proven to be useful, however. SNPs are a class of genetic markers used for characterizing allelic variation, genome-wide mapping and as a tool for marker-assisted selection [30]. Discovery of SNPs in tomato that are associated with resistance to Bacterial speck and race T1 of Bacterial spot has been described that involves a combination of data mining, estimation of intron position, *de novo* sequencing, and experimental verification [30]. This approach, data mining for SNPs utilized computer aided analysis of expressed sequence tags (ESTs) [31] . This in turn takes advantage of the availability of large tomato ESTs public databases. While some of the SNPs discovery in tomato has already begun [29, 31, 32], ongoing SolCAP research project (http://solcap.msu. edu/) aims to develop more than 1500 SNP markers, which are expected to be extremely useful for resistance breeding, particularly in tomato since there is very low level of genetic variation at molecular level [33]. In SNP, difference of a single base pair among the individuals is detected. Advancement in the DNA sequencing technology has made this possible to detect such differences more easily [34]. Currently, there are three different platforms Roche 454 GS-FLX sequencer, Illumina genome analyzer and SOLiD sequencer, which have revolutionized the DNA sequencing and is the basis of SNP markers discovery [34-36]. This technology has already been proven to be useful to detect the polymorphism in other plants [35-37], and is expected to be more useful in tomato. Information on SNP markers developed in tomato is available in solanaceae genomics network (www.sgn.cornell.edu). 

Currently there are 330,396 ESTs deposited in the tomato gene index database (http://compbio.dfci.harvard.edu/tgi/cgi-bin/tgi/T_release.pl?gudb=tomato), out of which 41,425 are unigenes- set of transcripts from the same transcription factor. The *Solanaceae* Genomics Network (SGN) at Cornell has 206,497 non-redundant EST sequences, out of which 34,829 are unigenes. Furthermore, 26,363 unigene sequences have been assembled out of 186,404 ESTs by MiBASE (http://www.kazusa.or.jp/jsol/ microtom/). It should be noted that 31,966 ESTs in MiBASE are associated with pathogen infection. This resource can be further mined for molecular markers for MAS. In addition, this genetic resource can facilitate the identification of resistant genes against various fungal diseases in tomato. 

In addition to EST information, a more global picture of the tomato defense network at the genome level will facilitate developing novel resistant varieties. The ongoing tomato genome sequencing project led by the International Solanaceae Genome Initiative (ISGI) will help make this possible. Tomato has a genome of approximately 950 Mb. A BAC by BAC sequencing strategy was proposed to sequence the 220 Mb gene-rich euchromatin [38]. As of June 13, 2009, 44% of this sequencing was reported to be complete (http://www.sgn.cornell.edu/about/tomato_sequencing.pl). The genomes of several plant species including *Arabidopsis* [39], rice [40] and poplar [41] have been fully sequenced. Specific defense networks in some of these plants, such as *Arabidopsis*, are beginning to be characterized. Evidence indicates that some of these defense pathways, such as the jasmonic acid and salicylic acid signaling pathways are conserved in plants. With the tomato genome sequence becoming available in the near future, the orthologs of characterized defense-related genes in *Arabidopsis* can be readily identified in tomato through data mining, as has been demonstrated in other plants [42]. Furthermore, availability of whole genome sequence of tomato would not only facilitate the localization of genes but also positional cloning as has been discussed in other species [43]. 

## FUNCTIONAL GENOMICS 

Determining the function of a set of resistance genes helps us understand the pathway leading to the resistance reaction of a host plant. Given the availability of relatively cheap microarray technology, a large number of genes can be assessed at once with respect to their expression in response to particular fungal disease. For instance, chitin is found in fungal cell walls but not in plants. Plant cells can perceive chitin fragments, and perception of this “signal” then leads to induction of defense response genes. Wan *et al*. [44] identified a LysM receptor-like protein (LysM RLK1) required for chitin signaling in *Arabidopsis*. Mutations in this gene blocked the induction of almost all chito-oligosaccharide-responsive genes and led to increased susceptibility to fungal pathogens. Exogenously applied chito-oligosaccharides enhanced resistance against fungal pathogens in the wild-type plants but not in the mutant [44] indicating that LysM RLK1 is essential for chitin signaling in plants and is involved in chitin-mediated plant innate immunity. In yet another study, potato cDNA microarrays were utilized to identify genes that are differentially expressed in the host during a compatible interaction with *P. infestans*. Out of 7,680 cDNA clones represented on the array, 643 were differentially expressed in infected plants as compared with mock-inoculated plants [45]. 

Disease-resistance reactions were found to be correlated with changes in cellular modifications and the infiltration of phenolic compounds at sites of potential pathogen penetration [46]. Activation of the phenylpropanoid pathway appeared to be a crucial component involved in pathogen growth restriction and tomato plant cell survival under stress conditions. The tomato *Cf* genes confer resistance to the fungal pathogen *Cladosporium fulvum* through recognition of secreted fungal *Avr* peptides. One of the protein kinases called Avr9/Cf-9 induced kinase 1 (ACIK1) was rapidly up-regulated in tomato upon elicitation by Avr9 [47]. Silencing of ACIK1 in tobacco resulted in a reduced hypersensitive response (HR) that correlated with loss of ACIK1 transcript. Moreover, virus induced gene silencing (VIGS- a process of interrupting gene expression by introducing the single stranded virus into plant cells), of LeACIK1 in tomato decreased *Cf-9*-mediated resistance to *C. fulvum* demonstrating the importance of ACIK1 in disease resistance. In addition to *Cf-9*, the gene *9DC* from the wild tomato species *S. pimpinellifolium* also confers resistance to strains of *C. fulvum* that secrete the *Avr9* protein. It was found that *9DC* and *Cf-9* were allelic by sequence comparison [48]. Suspension cultures of tomato cells that carry the *Cf-9* resistance gene were used in order to study biochemical mechanisms of resistance. Treatment of cells with various elicitors, except *AVR9*, induced expression of defense-related genes. On further analysis, it was found that induction of defense responses in *Cf9* tomato cells by the AVR9 elicitor is developmentally regulated and is absent in callus tissue and cell-suspension cultures [49]. In order to identify the genes conferring virulence in the pathogen and resistance in host plants, the yeast secretion trap is a potentially valuable technique. This technique was applied to study the interaction between tomato and *P. infestans,* revealing sets of genes encoding secreted proteins from both pathogen and host [50]. Other technologies that are being used in tomato includes Targeting Induced Local Lesions In Genomes (TILLING) was developed for targeting local mutations in the genome. In this method, a series of mutants can be identified are amplified PCR product resulted through mutation [51]. These series of mutants which could be generated either by insertional mutagenesis or by natural point mutation can be used for the study of function of particular genes. Its application in tomato is still being developed, which is discussed elsewhere in a review [1].

### Transcriptomics 

One of the most widely used functional genomics tools is transcript profiling. This can be conducted with various genomic tools, such as microarrays, serial analysis of gene expression (SAGE), and massively parallel signature sequencing (MPSS). Based on the extensive collection of ESTs, several microarrays have been developed for tomato: a cDNA-based microarray called Tom1 containing approximately 8,000 unigenes, a long oligonucleotide-based microarray named Tom2 containing approximately 11,000 independent genes (http://ted.bti.cornell.edu/), and an Affymetrix array containing probe sets for approximately 10,000 genes (http://www.affymetrix.com/products_services/arrays/specific/tomato.affx). Such arrays make it possible to compare gene expression changes in resistant and susceptible plants at a global scale. 

*Pseudomonas syringae* pv. tomato (*Pst*) is a bacterial pathogen. Effector proteins, which are injected into the plant cell through the type III bacterial secretion system (TTSS) play an essential role in the development of bacterial speck disease. Microarray experiments based on Tom1 were performed to investigate the molecular roles of TTSS effectors in disease formation in tomato [52]. A total of 306 genes were found to be differentially expressed in tomato plants infected with wild-type Pst strain DC3000 or a mutant lacking a functional TTSS. Many of the differentially expressed genes encode proteins associated with hormone response or hormone biosynthesis pathways, including two genes, LeACO1 and LeACO2 encoding the ethylene-forming enzyme ACC oxidase. Further genetic study revealed that effector proteins AvrPto and AvrPtoB promote enhanced disease resistance in tomato leaves partly by upregulating LeACO1 and LeACO2 involved in ethylene production.

In another microarray study, more than 15 functional classes of proteins and a large number of transcription factors and signaling components were identified in tomato challenged by *Xanthomonas lycopersicii* [53]. This study led to the development of new hypotheses about the molecular basis of recognition between AvrRxv and the corresponding resistance proteins, and set the stage for the dissection of signaling and cellular responses triggered in tomato plants by this avirulence factor. 

Transcript profiling can also be used to study the interaction of plants with beneficial organisms. For example, microarrays have been used to study why *Trichoderma hamatum*-inoculated tomato plants are more resistant to bacterial spot of tomato caused by *Xanthomonas euvesicatoria* compared with control plant [54]. In this study, 45 genes were found to be differentially expressed in the leaves of control tomato plants and the leaves of *T. hamatum*-inoculated tomato plants before they were inoculated with the pathogen. This work suggests that *T. hamatum* 382 actively induces systemic changes in the physiology and disease resistance of tomato plants through systemic modulation of the expression of stress and metabolism genes. 

### Proteomics as a Tool for Studying Disease Resistance

While large-scale transcript profiling is a powerful approach, its application may be limited in certain circumstances. For example, the increases in mRNA do not always correlate with an increase in protein levels. In addition, once translated, a protein may or may not be active, depending on post-translational modifications. Therefore, large scale analysis of proteins and metabolites, which are called proteomics and metabolomics respectively, are additional useful tools for studying tomato defense mechanisms against various fungal diseases. One such example is AtCTR1 in *Arabidopsis*, which is a Raf-like protein kinase that interacts with ETR1 and ERS, and negatively regulates ethylene responses. A gene LeCTR2 from tomato is similar to AtCTR1 and is involved in defense and stress responses [55]. Examination of protein–protein interactions between LeCTR2 and tomato ethylene receptors indicated that LeCTR2 interacts preferentially with ethylene receptors LeETR1 and LeETR2. 

A *Capsicum annuum* hypersensitive induced reaction protein1 (CaHIR1) was proposed as a positive regulator of hypersensitive cell death in plants. Overexpression of CaHIR1 in transgenic *Arabidopsis* plants conferred enhanced resistance against *Pst* [56]. A quantitative comparative proteome analysis using two-dimensional gel electrophoresis coupled with mass spectrometry was performed to identify altered protein accumulation. Out of 400 soluble proteins, 11 proteins were differentially-regulated by CaHIR1. Some of those proteins were glycine decarboxylase, Arabidopsis carbonic anhydrase, and copper/zinc superoxide dismutase. These proteins are involved in plant responses to biotrophic, hemi-biotrophic and necrotrophic pathogens [57]. Phosphorylation of proteins has long been reported to play a central role in signal transduction pathways in plant defense against pathogens. Changes to the phosphoproteome of *Arabidopsis *during the defense response to *Pst* DC3000 was investigated by Jones *et al*. [58]. They identified five proteins Rubisco, dehydrin, a putative p23 co-chaperone, heat shock protein 81 and a fibrillin, which are known to have potential phosphorylation sites as a part of plant basal defense system. The large subunit of Rubisco showed a significant difference between tissue undergoing the hypersensitive response and a basal defense response, which has been reported to be involved in stress tolerance [59]. 

Such interaction between proteins in *Arabidopsis *has also been reported between proteins from the enhanced disease susceptibility gene (*EDS1)* and the senescence associated gene 101 (*SAG101*) [60]. Together with phytoalexin-deficient-4 (*PAD4*), a known *EDS1* interaction, SAG101 contributes crucial signaling activity to EDS1-dependent resistance. In fact, interactions of EDS1 and its signaling partners in multiple cell compartments are important for plant defense signal relay. Systemin has been identified as a primary signal molecule involved in the local and systemic responses to pest attack [61]. In particular, engineered plants showed a number of differentially synthesized proteins that were identified by mass spectrometry approaches as polypeptide involved in protection from pathogens and oxidative stress. These proteomics tools have thus been used to identify proteins involved in resistance reaction [59].

Despite their utility, fewer studies of proteomics of tomato in the context of plant-pathogen interactions have been conducted. In contrast, a number of proteomic studies have been conducted to understand tomato fruit development [62] and resistance against environmental stresses such as water logging [59]. The proteomic study of tomato-pathogen interactions can be compounded by the presence of microbial proteins. This issue can be resolved by the availability of full tomato genome sequence and the genome sequence of infecting pathogens. 

### Metabolomics as a Tool for Studying Disease Resistance

Metabolites are organic compounds that are starting materials, intermediates, or end products of plant metabolism and often represent the end-products of gene expression and enzymatic activity. Up-regulation of expression of genes of metabolism is a frequently observed response of plants under stresses [63], indicating that metabolic changes play an important role in plant defense. Quantitative and qualitative measurements of all kinds of cellular metabolites, or metabolomics can yield a global view of the biochemical phenotype of an organism. Detection of those metabolites that exhibit significant variations in quantity and quality in control *vs*. pathogen-inoculated plants, or susceptible compared to resistant plants will provide novel insights about the functions of the compounds. A recent study has used this approach to investigate potato plant and *P. infestans* interactions. Using GC-MS, a total of 106 consistent peaks were detected, of which 95 metabolites were identified. Following *P. infestans* inoculation, the abundance of 42 metabolites were significantly changed, which were designated as pathogenesis-related [64]. Factor analysis of the abundance of the initial 106 metabolites identified four plant–pathogen interaction functions: (i) homeostasis; (ii) primary defense; (iii) secondary defense; (iv) collapse of primary and secondary defense responses. 

Plants produce a wide array of metabolites, many of which are not essential for plant growth and development and are thus called secondary metabolites. In addition to their important roles in human health, plant secondary metabolites are important in plant defense systems [65, 66]. Details about the use of metabolomics in different aspects of plant improvement including disease resistance is discussed by Bino *et al. *[67]. Flavonoids are one class of secondary metabolites, and comprise a large and diverse group of polyphenolic compounds [68]. Their genes have been used to engineer the flavonoid biosynthetic pathways in both model and crop plant species, not only from a fundamental perspective, but also to alter disease resistance. Bovy *et al*. [68] have recently reviewed the advances made in changing the flavonoid pathway in tomato, which covers the role of flavonoids in host reaction to the pathogens. Tomatoes are known to produce a wide array of secondary metabolites, many of which accumulate in the trichome [69]. Some wild tomatoes are known to produce a large amount of methylketone, which is reported to act as a defense compound. With the help of metabolomics, the gene responsible for methylketone biosynthesis was identified through the combination of transcript profiling and metabolomics.

In general, it is accepted that there is no single analytical method that can provide sufficient visualization of the entire metabolome due to the diversity of plant metabolites. The methods of metabolic profiling, therefore, must provide a compromise between the breadth of the metabolites that can be measured and the quality of the measurement [70]. These have been significant process in the field of metabolomics. While the use of metabolomics for studying tomato fungal diseases is still in its infancy, the method is likely to be used in conjunction with the development of other analytical tools in the future. 

### Gene Silencing

Finally, relatively new methods collectively called gene silencing have proved to be valuable tools for determining and confirming the function of genes in tomato. Gene silencing is a technique in which activity of gene is switched off. Switching off of a gene could result either reduced level of transcription (process of synthesis of mRNA from genomic DNA) or reduced levels of translation (the process of protein synthesis on the basis of codons in the mRNA) from specific genes [71]. In this process, an enzyme complex ‘Dicer’ plays a key role. Dicer has two catalytic sites of an RNase III-family that cleave double stranded (dsRNA) at two locations that are separated by about 21 nucleotides. The cleaved dsRNA fragment has an overhang of one or two nucleotides at the 3′ end and a phosphate at the 5′ end. This fragment is separated into single-strand RNAs and one strand can serve as a guide for RNA-induced silencing complex (RISC). The RISC is attached to a specific sequence in an mRNA, and the mRNA is either cleaved or its translation is suppressed [72]. Hence specific gene can be targeted with this technique to determine the function of gene(s). This can be achieved through introduction of a small complementary single stranded RNA (sRNA) in a sense or anti-sense orientation, or by introducing a dsRNA by *Agrobacterium*-mediated or particle bombardment methods. The gene silencing approaches are used to determine the function of particular genes hence this is yet another important tool in functional genomics.

While there are a few studies related to virus disease resistance in tomato using gene silencing approach [73, 74], it is lacking in fungal diseases resistance. It has been used in *Nicotiana benthamiana* to suppress expression of *LePP2Ac* gene, which encodes a catalytic sub-unit of the heterotrimeric protein phosphatase 2A (PP2A). The *LePP2Ac*-silenced plants had greatly decreased PP2A activity, constitutively expressed many pathogenesis-related genes, and developed localized cell death in stems and leaves [75]. The plants were resistant to *Pseudomonas syringae* pv. *tabaci* and showed an enhanced hypersensitive response to effector proteins from fungal pathogen of tomato *Cladosporium fulvum*. This provided an insight of possibility of using this approach in developing resistance to fungal diseases in tomato but yet to exploit the approach. 

## COMPARATIVE GENOMICS FOR STUDYING DISEASE RESISTANCE 

Comparative genomics is the comparison and analysis of genomes from different species. The purpose is to gain a better understanding of structural and functional genomics of one species with respect to others [76]. Many different features are considered when comparing genomes of different species such as sequence similarity, gene location, the length and number of exons, number of introns, and conserved sequences. With this information, at least tentative gene identification in the genome of a species is possible. It also provides a tool to utilize the exponentially increasing sequence information from model plants to clone agronomically important genes from less studied plant species. Kamoun and Smart [77] have gained insights about the possible ways to use the genomics for the understanding of biology of *P. infestans* and in the development of LB resistance in tomato. A local RGA approach using genomic information from tomato to isolate *R3a*, a potato gene that confers race-specific resistance to the LB pathogen *P. infestans* was used. *R3a* is a member of the *R3* complex locus located on chromosome 11 of tomato [78]. Comparative analyses of the *R3* complex locus with the corresponding *I-2* complex locus in tomato suggested that this is an ancient locus involved in plant innate immunity against fungal pathogens.

Comparative genomics approaches have also been used for investigating mechanisms used by pathogens to avoid the host defenses. Host specific virulence candidate genes were identified in *Pst* by searching for genes whose distribution among natural *Pst *isolates was associated with hosts of isolation. A total of 91 strains, isolated from 39 plant species, were analyzed by DNA microarray-based comparative genomic hybridization against an array containing 353 virulence-associated genes. Individual genes, significantly associated with strains, were identified and isolated from tomato [79]. Although they used bacteria in this study, the approach may also be applicable in fungi. The availability of the complete genome sequence of *Arabidopsis*, and the extensive EST resources in tomato, comparative genomic studies of important plant defense pathways such as the phenylpropanoid pathways can be further investigated across species [80]. Microarray analysis also provides the ability to perform possibilities for comparative analyses of global changes in specific transcripts in response to fungal infection [81]. 

## GENETIC TRANSFORMATION FOR DISEASE RESISTANCE

Despite the use of sophisticated and intensive crop-protection measures, particularly in industrialized countries, pathogens still cause substantial losses in tomato yield throughout the world. While conventional breeding has played a pivotal role in developing resistant varieties of tomato, there are several diseases which have yet to be addressed. Genetic transformation has been proved to be extremely useful tool for the development of disease resistance in tomato. In fact, tomato is one of the first plant species to use this technology in early 1980s [82, 83]. Since then, much progress has been made using genetic transformation technology and it can be considered as a key tool for the understanding of plant biology including functional genomics.

### Over-Expression of Transgenes

Advances in genetic transformation technology has provided the foundation for an environmentally friendly and economically viable approach for disease management in modern agriculture [84]. However, before selecting any gene for introduction into plant, it is important to understand the biology of host-pathogen interaction. Many pathogens have co-evolved with their host plants and have developed many ways to overcome plant defense responses. To effectively make use of components of the plant defense system, it is important to understand the co-evolution of plants and their pathogens at the molecular level. All bacterial plant pathogens appear to produce an effector molecule that triggers disease resistance responses when a plant carries the corresponding resistance gene. Transgenes are often selected based on this assumption.

The enzyme stilbene synthase is found in several plant species and synthesizes the stilbene phytoalexin trans-resveratrol using substrates commonly present in plants. The formation of stilbenes is considered to be a component of general defense mechanisms against pathogens in plants [85, 86]. Two genes from the grapevine (*Vitis vinifera*) coding for stilbene synthase were transferred to tomato cv. Vollendung using *Agrobacterium*-mediated transformation [87]*.* The accumulation of the phytoalexin trans-resveratrol was detectable shortly after fungal inoculation resulting in a significant increase in the resistance of transgenic tomato to *P*. *infestans.* A similar accumulation of resveratrol occurred after inoculation with *Botrytis cinerea *and *A. solani*. These results indicate that expression of the transgene in the host plant enhanced resistance to the multiple diseases. T-DNA insertion mutagenesis has also been used to obtain tomato plants resistant to LB. Plants thus mutagenized were inoculated with a mixed *P. infestans* population combining isolates from the field. Among the primary transformants (T0) and T1 generation, plants with increased resistance to LB [88] were obtained at a frequency of 0.1%.

In another study [89], genes coding for a maize β-glucanase (M-GLU), and a *Mirabilis jalapa* antimicrobial peptide (*Mj*-AMP1) were separately introduced into tomato cv. Sweet Chelsea *via Agrobacterium*-mediated transformation. Plants of selected transgenic lines were inoculated with *A. solani*, and scored for disease resistance. Compared to control plants, two transgenic lines carrying either an *M-GLU* or *Mj-AMP1* showed enhanced resistance to EB disease indicating the effectiveness of transgenes in developing resistance to EB.

Expression of the constitutively active form of mitogen-activated protein kinase (MAPK) driven by a pathogen-inducible promoter in potato plants has also been reported to enhance resistance to EB and LB [90]. In these transgenics, pathogen attack induced expression of MAPK was closely followed by activation of NADPH oxidase, and resulted in a rapid hypersensitive reaction. Since potato and tomato have very similar genomes, it is expected that this gene might be effective in tomato to enhance resistance to these two fungal diseases. Recently, a major LB resistance gene (RB) was identified in the wild potato species *Solanum bulbocastanum* and the full-length gene was integrated into cultivated potato using *Agrobacterium*-mediated transformation [91]. The *RB*-containing transgenic plants were challenged with *P. infestans* under favorable conditions for LB in greenhouse experiments. All transgenic lines containing *RB* exhibited strong foliar resistance.

In yet another study, a cDNA of wheat oxalate oxidase (*OxO*) was expressed in tomato. Twenty-six transgenic tomato lines were obtained that expressed a 124-kDa protein with varying levels of OxO activity, producing different amounts of oxalic acid *in vitro*. Most of these transgenic lines showed reduced LB symptoms compared to controls in a detached leaf assay [92]. Interestingly, some of the plants were also more resistant to *Botrytis cinerea* and *Sclerotinia sclerotiorum*, which are also important fungal diseases of tomato.

VW is another fungal disease of tomato that causes significant yield losses, although its presence has been reported to be sporadic in various tomato growing areas. Resistance to this disease was achieved by introducing an acidic endo-chitinase gene (*pcht28*) isolated from *S. chilense* [93]. Transgenic plants demonstrating a high level of constitutive expression of *pcht28* and correspondingly high chitinase enzyme activity significantly increased tolerance to VW as compared to the non-transgenic plants as measured by decreased foliar symptoms, vascular discoloration, and decreased vascular discoloration index. Following a similar approach, plants resistant to FW have also been developed [94]. There is a lot of work yet to be done to improve the resistance in tomato for other fungal diseases although basic information and potential transgenes have been identified in other plant system such as *Arabidopsis*.

## CONCLUSION 

Molecular markers and QTL analysis work performed so far has been useful for locating the resistance gene(s) on the genome of tomato and to carry out marker-assisted selection for some of the fungal diseases. It has provided foundation for fine-mapping and advancing the molecular breeding for others. As we will have the whole genome sequence available of tomato, it will be helpful to advance the molecular breeding by facilitating the positional cloning as well as marker-assisted selection. This will also be an instrumental in determining the function of disease resistance genes available in the tomato genome. Complexity of disease resistance mechanism will be dissected relatively more easily as we decipher the function of genes involved in such pathways. Connecting the dots of current efforts in genomics, metabolomics and proteomics with the whole genome sequence is expected take our understanding to a novel height.

## ABBREVIATIONS

BAC: = Bacterial artificial chromosome
BC: = Backcross
CAPS: = Cleaved amplified polymorphic sequence
EB: = Early blight
EST: = Espressed sequence tag
FW: = Fusarium wilt
LB: = Late blight
MAS: = Marker-assisted selection
NIL: = Near-isogenic lines
PM: = Powdery mildew
QTL: = Quantitative trait loci
RAF: = Randomly amplified DNA fingerprinting
RFLP: = Restriction fragment length polymorphism
RGA: = Resistance gene analog
SCAR: = Sequence characterized amplified region
SGN: = Solanceae genomics network
SLS: = Septoria leaf spot
SNP: = Single nucleotide polymorphisms
SSR: = Simple sequence repeat
VW: = Verticilium wilt
